# Proteomics: Progress and Promise of High-Throughput Proteomics in Chronic Kidney Disease

**DOI:** 10.1016/j.mcpro.2023.100550

**Published:** 2023-04-17

**Authors:** Pascal Schlosser, Morgan E. Grams, Eugene P. Rhee

**Affiliations:** 1 Department of Epidemiology, Johns Hopkins Bloomberg School of Public Health, Baltimore, Maryland, USA; 2 Division of Precision Medicine, Department of Medicine, New York University, New York, New York, USA; 3 Nephrology Division and Endocrine Unit, Massachusetts General Hospital, Boston, Massachusetts, USA

**Keywords:** Proteomics, chronic kidney disease, high-throughput, causal inference, Mendelian randomization, pQTL

## Abstract

Current proteomic tools permit the high-throughput analysis of the blood proteome in large cohorts, including those enriched for chronic kidney disease (CKD) or its risk factors. To date, these studies have identified numerous proteins associated with cross-sectional measures of kidney function, as well as with the longitudinal risk of CKD progression. Representative signals that have emerged from the literature include an association between levels of testican-2 and favorable kidney prognosis and an association between levels of TNFRSF1A and TNFRSF1B and worse kidney prognosis. For these and other associations, however, understanding whether the proteins play a causal role in kidney disease pathogenesis remains a fundamental challenge, especially given the strong impact that kidney function can have on blood protein levels. Prior to investing in dedicated animal models or randomized trials, methods that leverage the availability of genotyping in epidemiologic cohorts—including Mendelian randomization, colocalization analyses, and proteome-wide association studies—can add evidence for causal inference in CKD proteomics research. In addition, integration of large-scale blood proteome analyses with urine and tissue proteomics, as well as improved assessment of posttranslational protein modifications (*e.g*., carbamylation), represent important future directions. Taken together, these approaches seek to translate progress in large-scale proteomic profiling into the promise of improved diagnostic tools and therapeutic target identification in kidney disease.

## Highlights

• Proteomics permits the high-throughput analysis of the blood proteome in large cohorts.
• Mendelian randomization, colocalization analyses, and proteome-wide association studies can add evidence for causal inference in kidney disease proteomics research.
• Levels of testican-2, TNFRSF1A, and TNFRSF1B are associated with kidney prognosis.
• Taken together, these approaches seek to translate progress in large-scale proteomic profiling into the promise of improved diagnostic tools and therapeutic target identification in kidney disease.

## Graphical Abstract

**Figure:**
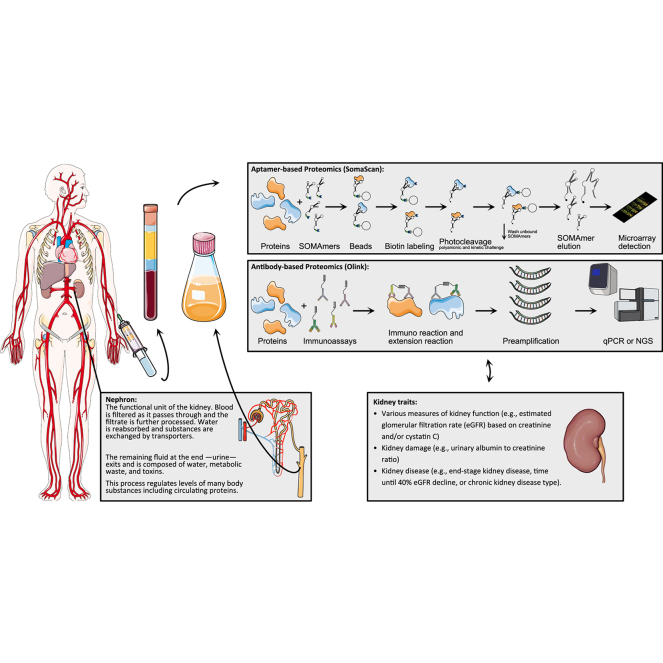

## In Brief

Population genetics methods leverage advances in large-scale proteomic profiling to improve the accuracy of diagnostic tools and the identification of therapeutic targets for kidney disease.

**Fig. 1:**
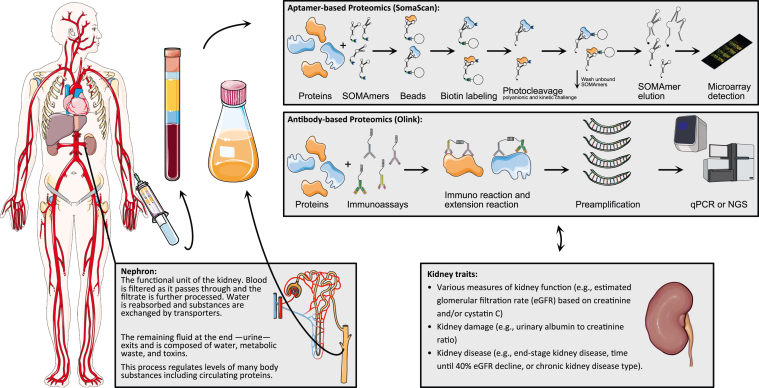
**Overview of proteomics in epidemiological studies on kidney diseases.** *Icon credit: Servier Medical Art by Servier (licensed under a Creative Commons Attribution 3.0 Unported License).*

Assays of select peptides and proteins in blood and urine are fundamental to the practice of nephrology. For example, blood levels of cystatin C are routinely used to assess kidney filtration (*i.e*., estimated glomerular filtration rate, eGFR), whereas blood levels of complement factors and parathyroid hormone can be used to characterize the cause and metabolic consequences of kidney disease, respectively. Similarly, urine levels of albumin are used to gauge the severity of kidney damage and can also provide clues about underlying kidney disease etiology. Given this background, proteomics has been increasingly employed in kidney disease with the goal to improve diagnosis and risk prognostication and to provide pathophysiologic insight (Fig. 1). In this Perspective, we provide a focused update on the progress of proteomics in kidney disease, as well as outline some areas of promise and future direction.

Two key caveats warrant mention up front. First, our Perspective prioritizes studies that have utilized multiplexed methods using aptamers or nucleotide-labeled antibodies for specific protein capture, followed by detection and quantitation based on nucleic acid oligomerization (1, 2). The high throughput enabled by these methodologies has catalyzed proteomic profiling in several large, epidemiologic cohorts. The aptamer-based SomaScan platform has developed from ∼1000 to more than 7000 aptamers over the past decade. Similarly, O-link continuously extended and combined their targeted immunoassay panels and covers nearly 3000 proteins with the current Olink Explore 3072 platform. Both can be applied in a high-throughput design enabling even biobank scale projects. Prime examples include two studies that identify genetic determinants of proteins in 35,559 Icelanders (4907 aptamers) (3) and 54,306 UK Biobank participants (1463 proteins) (4), respectively. The second key caveat is that we prioritize proteomic analyses of blood, the biofluid most frequently subject to testing in the clinic and that has been assayed in the large majority of epidemiologic studies to date. Analyses of urine and kidney tissue itself are of potentially high relevance to kidney disease, but large-scale results in these areas are pending.

Before focusing on proteomic analyses of blood, we would be remiss if we did not acknowledge the major insights that have been gained by proteomic analysis of kidney tissue using other methodologies. For example, LC-MS has been utilized to identify a number of antigens that underlie the antibody–antigen immune complexes within the kidney that characterize idiopathic membranous nephropathy, an autoimmune kidney disease. In a landmark study, Beck *et al.* (5) probed human kidney extracts with sera from individuals with membranous nephropathy and then used LC-MS of the reactive protein band to identify the M-type phospholipase A(2) receptor as the most common target antigen for circulating antibodies in patients with membranous nephropathy. Tomas *et al.* (6) used a similar approach to identify thrombospondin type-1 domain-containing 7A as a target antigen in some individuals with anti-PLA2R1-negative disease. Most recently, Sethi *et al.* (7) has utilized laser microdissection of kidney biopsy samples followed by LC-MS to identify several additional novel antigens that underlie membranous nephropathy: exotosin 1, exotosin 2, NELL1, Sema3B, and PCDH7. Together, these discoveries have transformed clinical care, providing more specific disease designations and yielding new tools for diagnosis and monitoring the response to therapy, *i.e.*, levels of circulating antibodies specific to these newly discovered antigens. Ultimately, the application of proteomics to much larger patient populations seek to yield insights of similar import but generalizable to more common forms of kidney disease.

## Proteomic Studies of Kidney Disease in Epidemiologic Cohorts

For the purpose of kidney disease research, analyses of blood-based proteomic data in large patient cohorts often fall into one of three categories or some combination thereof: (1) cross-sectional association of protein levels with kidney function, (2) identification of proteins at a baseline time point that are associated with the future onset of chronic kidney disease (CKD), or (3) identification of proteins at a baseline time point that are associated with CKD progression, *e.g.*, progression to end-stage kidney disease (ESKD). These studies generally consider all CKD etiologies together or look specifically at diabetic kidney disease (DKD), the most common cause of ESKD worldwide.

Ngo *et al.* (8) began with a cross-sectional examination of arterial to renal venous gradients for >1300 proteins among 22 individuals who had undergone invasive sampling. They found that several dozen proteins demonstrated a significant change from artery to vein, with the large majority demonstrating a decrement across the kidney, consistent with net clearance by the kidney (whether *via* glomerular filtration, tubular secretion, or renal catabolism). Interestingly, a few proteins increased from artery to vein, consistent with net release by the kidney. Testican-2, a protein encoded by *SPOCK2*, had the most significant augmentation from artery to vein, increasing in all study participants. Next, the authors examined plasma proteomics data from two large population-based study cohorts—the Jackson Heart Study and the Framingham Heart Study—spanning ∼3500 individuals with normal kidney function. In the discovery cohort, >40% of the >1000 measured proteins had a significant cross-sectional association with kidney function, as defined by eGFR, whereby lower eGFR signifies worse kidney function. The majority of these proteins were inversely associated with eGFR (lower eGFR, higher protein level). Consistent with the data from renal arteriovenous sampling, testican-2 had the single strongest positive association with baseline eGFR (higher eGFR, higher protein level). Further, higher testican-2 levels at baseline were associated with lower rate of eGFR decline and lower risk of incident CKD in these cohorts.

In a follow-up study, Wen *et al.* (9) found that higher testican-2 levels were associated with a lower risk of CKD progression, defined as incident ESKD, across ∼8000 individuals spanning three study cohorts enriched for prevalent CKD and its risk factors: the Chronic Renal Insufficiency Cohort (CRIC), the African American Study of Kidney Disease and Hypertension, and the Atherosclerosis Risk in Communities studies. Microscopy of human kidney samples demonstrated glomerular expression of testican-2, and single-cell RNA sequencing showed that kidney expression of *SPOCK2* occurs exclusively in podocytes. The *in vivo* function of testican-2 is unknown. It is a 424 amino acid secreted glycoprotein predicted to form part of the extracellular matrix, and *in vitro*, it can increase glomerular endothelial cell tube formation and migration and increase Matrix metalloproteinases-2/matrix metalloproteinases-9 activity (8). Together, these studies highlight testican-2 as a potential podocyte-derived marker of kidney health and prognosis. However, more work is required to elucidate renal testican-2 biology and to determine whether it plays a functional role in kidney protection.

Whereas testican-2 is associated with better kidney health, most proteomic studies to date have highlighted markers associated with worse kidney prognosis. For example, Grams *et al.* (10) quantified the association between ∼4800 proteins and a composite outcome of ESKD or decline in eGFR by ≥50% among >9000 participants in the Atherosclerosis Risk in Communities study—in this analysis, all 13 significant associations were for proteins associated with increased risk of CKD progression. Nine of these associations were validated in ∼4000 participants of the CRIC study and the African American Study of Kidney Disease and Hypertension, highlighting both known and novel biomarkers of kidney risk. The association between TNFRSF1A and TNFRSF1B and CKD progression, in particular, has been repeatedly reported in the literature (11, 12, 13). This includes studies utilizing targeted assays for these molecules (see Liu, *et al.* (14) for a recent systematic review of targeted biomarker studies in CKD), as well as other proteomic analyses. For example, Niewczas *et al.* (15) began with the hypothesis that chronic inflammation contributes to the development of ESKD in diabetes and thus looked at a subset of 194 inflammatory proteins in >500 individuals with DKD drawn from the Joslin Diabetes Study and the Pima Indian Kidney Study. The authors found that a panel of proteins enriched for TNF Receptor Superfamily receptors, including TNFRSF1A and TNFRSF1B, were associated with 10 years risk of DKD progression. Unlike testican-2, all of these proteins were found to have nonkidney sources, indicative of the potential deleterious impact of systemic inflammation on the kidney.

Building out from the hypothesis-driven analysis of inflammatory proteins, the Joslin Diabetes Study and Pima Indian Study investigators have published four additional key studies considering other portions of the proteome assayed in their study cohorts. Three of these studies analyzed other predefined subsets of proteins such as axon guidance pathway proteins that were selected based on miRNA profiling (16), candidate proteins identified as protective factors against progression to ESKD (17), and TGF-β pathway–related proteins (18), and one study examined the remaining 795 proteins in an “untargeted” analysis (19). In sum, these analyses highlight >40 proteins associated with DKD progression. Interestingly, the proteins identified in the hypothesis-free study had similar odds ratios and *p*-values of association for ESKD as the proteins identified in the hypothesis-driven analyses, underscoring the value of an untargeted approach to proteomic discovery. To what extent these findings are specific to DKD or can be generalized to other causes of CKD requires further analysis—as with TNFRSF1A and TNFRSF1B, many of the associations identified in Joslin and Pima studies have been corroborated by proteomic associations with eGFR decline and CKD progression among individuals without diabetes. Ongoing proteomics analyses by the NIDDK CKD Biomarkers Consortium focused on the CRIC study, in which ∼50% of the participants have diabetes, should provide some insight into this question of specificity.

## The Major Impact of GFR in the Analysis and Interpretation of Proteomics Data in Kidney Disease

**Fig. 2:**
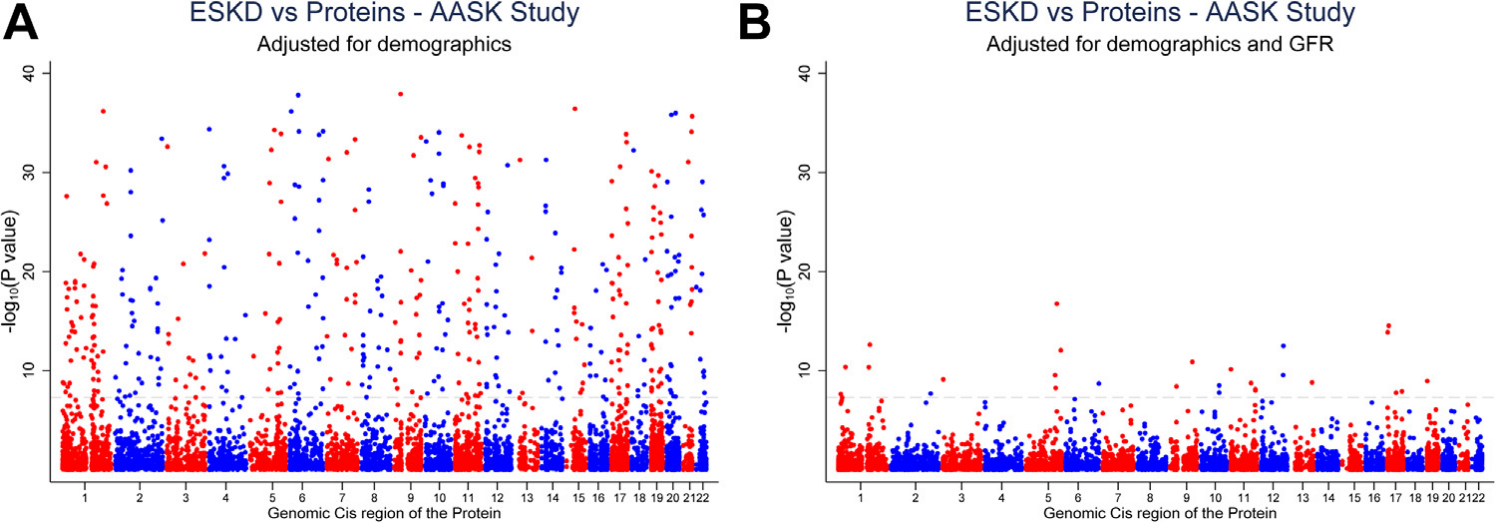
**The effect of adjustment for (measured) GFR in studies of proteomics data and ESKD.** Here, we fitted Cox proportional hazards models for 7,285 proteins quantified in 703 participants of the AASK study. Protein abundance was log-transformed and adjusted for demographics included age, sex, and the randomization group. GFR, glomerular filtration rate; ESKD, end-stage kidney disease; AASK, the African American Study of Kidney Disease and Hypertension; ESKD, end-stage kidney disease.

Cross-sectional analyses of proteins with GFR often demonstrate many significant correlations, especially inverse correlations whereby lower GFR is associated with higher blood protein levels. In turn, prospective analyses that seek to identify proteomic predictors of future GFR loss or CKD progression invariably use statistical models that adjust for baseline GFR, as well as other standard risk factors for CKD including proteinuria, age, gender, diabetes status, and hypertension. Of these, the GFR adjustment has the largest impact (Fig. 2). This adjustment is clearly appropriate if the goal is to identify novel filtration biomarkers, but it is important to understand the potential limitations of adjusting for GFR in this context. First, both eGFR and directly measured GFR.(*e.g*, *via* administration and measurement of clearance of an exogenous compound) suffer from varying degrees of imprecision. Further, GFR does not capture the full breadth of kidney function that can impact the proteome, such as tubular absorption, metabolism, or secretion. Thus, adjusting for GFR does not exclude residual confounding attributable to differences in other aspects of kidney function. Finally, adjusting for GFR has the potential to obscure biologically meaningful signals. For example, a protein might have an inverse correlation with GFR because it is nephrotoxic and causes GFR decline, rather than simply accumulating as a bystander because of reduced renal clearance. This is an important consideration, as the proteome subsumes a broad range of biologic domains including inflammation, thrombosis, metabolism, cell growth, and differentiation, with potential relevance to kidney disease pathogenesis. Circulating cytokines (*e.g.*, TNFRSF1A and TNFRSF1B), enzymes, and hormones are all attractive as potential causal factors in the onset and propagation of kidney disease.

## Assessment for Causal Relationships Between Proteins and Kidney Disease: Integration of Proteomics and Genomics in Epidemiologic Cohorts

For biomarker identification, understanding whether there is evidence for causation in proteomic screens (*e.g*., whether proteins cause low GFR or vice versa) is not essential. However, understanding causation and, more specifically, the direction of causation is crucial for elucidating underlying biology and the evaluation of putative drug targets. Prior to investing in dedicated animal models or randomized trials, other methods that take advantage of the availability of genotyping in epidemiological cohorts can provide additional evidence for causal inference in CKD proteomics research.

As a starting point, one needs to assess the associations between protein levels and genetic variants within a study cohort where both have been assayed.

This can be done in Genome-Wide Association Studies (GWAS), which consider genetic variants across the genome as the independent variable and (usually log-transformed) protein levels as the dependent variable. Genetic variants found to be significantly associated with protein levels are referred to as ‘pQTLs’, or protein Quantitative Trait Loci, and are classified as *cis* if the genetic variants are located in the protein coding region ±500 kilobases and as *trans* if they are located outside this region. The close proximity between gene and encoded protein expression can translate into pQTLs that explain substantial proportions of the variance of circulating protein levels (3, 20, 21, 22, 23, 24, 25). Several biobank-scale studies have performed GWAS of protein levels and made the summary statistics (*e.g*., beta coefficient, standard errors, *p*-values) of the identified pQTLs publicly available. If a protein is genetically regulated and pQTLs explain a significant proportion of protein variance, we can utilize these pQTLs for causal inference.

### Mendelian Randomization to Evaluate for Causality in Protein–Kidney Function Analyses

**Fig. 3:**
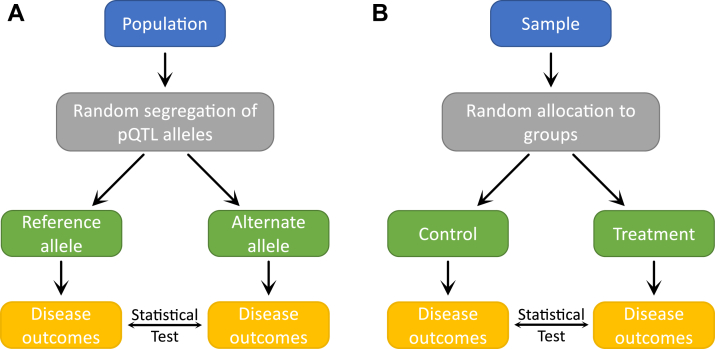
**Conceptual parallels of Mendelian randomization (*A*) and a randomized controlled trial (*B*).** Figure adapted from Figure 1 in Sanderson, et al. 2022. pQTL, protein Quantitative Trait Loci.

Mendelian randomization (MR) analyses make use of summary statistics from GWAS of protein levels as well as GWAS of kidney traits (*e.g.*, eGFR, blood urea nitrogen, albuminuria, eGFR decline) to evaluate for evidence of causal associations. To assess whether higher protein levels cause low GFR, MR uses pQTLs as instrumental variables (26). An instrumental variable is a variable associated with an exposure (the protein) that is not associated with the outcome (*e.g.*, kidney function or ESKD) through any other pathway. To support this assumption, we typically restrict instruments to *cis*-pQTLs—that is, if genetic variation within the protein coding region affects the outcome, it is likely through its effect on levels of the encoded protein. However, sensitivity analyses might allow us to additionally incorporate *trans*-pQTLs as instruments. MR analyses have been described as naturally occurring randomized trials, where the random assignment of pQTL alleles at birth leads to higher/lower protein levels across the lifespan, which may lead to a difference in disease outcomes (*e.g.*, GFR or ESKD) (Fig. 3) (26, 27). However, MR analyses are predicated on several strong assumptions, most of important of which is the assumption of no pleiotropy.

An instrumental variable is considered *pleiotropic* if it is associated with multiple phenotypes. In the example of proteins and kidney function, this can occur when a pQTL influences another phenotype (*e.g.*, blood pressure or another protein). If the other phenotype additionally has an effect on the outcome (*e.g.*, kidney function), this can lead to biased effect estimates in the MR analysis. The possibility that neighboring proteins might be coregulated with the protein of interest is an important consideration. Pairing MR with colocalization analysis provides one approach to address this potential confounding by linkage disequilibrium (28, 29). Colocalization analyses estimate a Bayesian posterior probability for the hypothesis of the same causal genetic variant underlying two GWAS signals, and two signals are said to be colocalizing if said probability is high.

### Two-Sample MR

MR can be conducted using individual patient data within a study cohort that contains data on genotypes, proteomics, and the outcome of interest (one-sample MR). However, two sample MR is less susceptible to overfitting and allows use of different cohorts, each of which may be missing one of these data types. Pairing two cohorts can also increase power by allowing for the largest sample size possible. For example, pQTL summary statistics from a large European ancestry population (deCODE + Icelandic Cancer Project, N= 35,559) (3) can be paired with a European ancestry GWAS of eGFR from the CKD Genetics Consortium (N = 1,004,040) (30). This approach relies on the assumption of matching linkage disequilibrium, meaning that the two GWAS should have the same underlying ancestry.

Importantly, the power of MR is limited by the proportion of the variance explained by the genetic variants. Currently, approximately 9.8% of the variance in eGFR appears to be genetically regulated (30); variance explained varies by protein but can be as high as 76.1% (3). Another current major limitation is the lack of ancestral diversity in existing GWAS, as well as sparse tissue specific protein GWAS (variation in blood protein levels may not reflect variation at the site of action, for example within the kidney). These limitations highlight why MR cannot replace animal models or randomized controlled trials; however, MR allows for a scalable approach to study hundreds of potential targets from cross-sectional associations (10, 31), building an improved foundation for follow-up experiments. In one recent study, the use of *cis* MR combined with colocalization analysis identified 111 putatively causal effects between 65 proteins and 52 disease-related phenotypes. In an evaluation of data from historic drug development programs, target-disease pairs with MR and colocalization support were more likely to have received agency approval when compared to targets without this genetic support (29).

In a kidney-specific analysis, Matias-Garcia *et al.* (31) assessed the bi-directional MR association of plasma proteins and eGFR. The authors found a positive causal effect of eGFR on testican-2; that is, genetic variants associated with higher eGFR were also associated with higher testican-2. These analyses also found suggestive evidence for a causal effect in the other direction, *i.e.*, for the proteins melanoma inhibitory activity, carbonic anhydrase III, and cystatin-M on eGFR. However, a causal effect was not observed for testican-2 levels on eGFR. Arguably, this reduces the likelihood that testican-2 plays a causal role in CKD pathogenesis, but importantly does not rule it out—the negative MR result could simply reflect that the genetic instruments for testican-2 are weak, with the top pQTL only explaining 2.2% of variance in blood testican-2 levels. Yu *et al.* (32) presented additional support for specific proteins implicated in the eGFR to protein direction. Genetic variants associated with eGFR cannot be classified into *cis* and *trans* QTLs since there is no inherent *cis*-region for eGFR. Instead, the authors utilized summary statistics from a GWAS of eGFR to combine millions of genetic variants in a polygenic risk score (PRS) (33). The PRS explained up to 9.4% of the eGFR variance, and they identified 132 proteins that were associated with the PRS, again including a positive association of eGFR and testican-2.

### Proteome-Wide Association Studies

Genetic Proteome-Wide Association Studies represent an especially streamlined approach to screen hundreds of proteins simultaneously (21). Here, the *cis*-genetic variation of proteins with significant heritability is utilized to build regression models to genetically impute protein levels and associate the genetically imputed levels with an outcome of interest. This can be placed into a methodologic equivalence with two-sample MR (34). The main difference from traditional MR is that instead of restricting the analysis to independent instrumental variables, penalized regression models that weight the potentially dependent instrumental variables in the *cis* region are used. Grams *et al.* identified LMAN2 as putatively causing lower eGFR. LMAN2 is widely expressed across tissues and is believed to participate in intracellular glycoprotein trafficking and quality control—how it might contribute to kidney disease requires further study. While MR and Genetic Proteome-Wide Association Studies can draw important inference when significant, it is important to recognize that besides the incorporated assumptions, they are also statistically demanding. Many reasons can lead to negative MR findings, *e.g.*, if only a small proportion of protein variance is explained by genetic instruments or if the true causal effect is contained in some cell type and not reflected in the blood proteomics that usually underly the pQTLs.

## Future Directions

Although this Perspective has focused on recent blood-based proteomic studies of kidney disease utilizing aptamers or nucleotide-labeled antibodies, we anticipate that ongoing and future large-scale analyses of urine will be similarly fruitful. Analysis of urine, using both targeted assays as well as other proteomic approaches, particularly MS-based, has been a productive area of emphasis in nephrology research for many years. For example, Mischak *et al.* have utilized capillary electrophoresis-MS to examine the association between urinary peptides and CKD. From a biomarker perspective, peptides (∼2–50 amino acids in length) have some advantages over full-length proteins, as they undergo glomerular filtration, and their measurement is more reproducible. Over the course of several studies, these investigators have found that a combination of 273 urinary peptides may be of higher utility than standard metrics for detection and prediction of CKD (35, 36, 37). Major components of this “CKD273 classifier” include collagen fragments, perhaps indicative of accumulation of intrarenal extracellular matrix, along with fragments of blood-derived proteins involved in inflammation and tissue repair. More targeted studies of urine have highlighted urinary markers of tubular cell injury (KIM-1 and α-1 microglobulin) (38, 39) as risk markers for progression of kidney disease. Newer, higher throughput methodologies have the potential to increase the scope of these studies, enhancing statistical power, extending generalizability, and enabling integration with genetics. Further, if conducted in cohorts that have already undergone profiling of blood, these studies will have the opportunity to conduct paired analyses of urine and blood protein levels.

In addition to performing assays of blood and urine, clinicians sometimes perform kidney biopsies in patients with kidney disease in order to obtain tissue for histologic examination, immunofluorescence, and even electron microscopy. Biopsies are usually not performed for the most common causes of kidney disease, *e.g*., diabetes and hypertension, or when there is clear genetic etiology of kidney disease such as autosomal dominant polycystic kidney disease. Instead, a biopsy is usually performed to make the diagnosis and guide treatment when the cause of kidney disease is unknown and/or a glomerular disease such as membranous nephropathy or lupus nephritis is suspected. Transplanted kidneys are also frequently subject to biopsy to assess for immune rejection. In these cases, biopsies provide a wealth of information, including severity, chronicity, and specificity about which compartments of the kidney are injured, for example the glomerulus, tubulointerstitium, or vasculature. A proteomic analysis of blood in a cohort of individuals that have undergone kidney biopsy would provide the opportunity to identify specific protein signatures of distinct pathologic findings, including specific diagnoses or more generalized patterns (*e.g.*, tubulointerstitial fibrosis). Of course, proteomic analysis of the kidney biopsy tissue itself is also valuable, as highlighted with the initial discussion of antigen discovery in membranous nephropathy. To date, however, aptamers or nucleotide-labeled antibodies have not been utilized for tissue samples at scale. The Kidney Precision Medicine Project, a major ongoing effort funded by the NIDDK, aims to collect kidney biopsy samples from individuals with common causes of both chronic and acute kidney disease, with the goal to generate single cell transcriptomics, metabolomics, and regional proteomics (laser microdissection followed by LC-MS) alongside clinical, pathological, and imaging data (40, 41).

In the context of MR, the underrepresentation of African Americans and other ancestries in genetic studies is a current limitation for causal inference. The ancestries of the proteomic and kidney function GWAS that are utilized for MR need to be aligned due to ancestry-specific differences in allele frequencies of genetic variants. While several studies have shown replicating effects between ancestries (8, 10), the differences in allele frequencies or exposure to different environmental factors might lead to differences in power and hence which causal effects are in a detectable range. Fortunately, several ongoing large-scale efforts, such as the Million Veterans Program (42) and the All of Us Research Program (43), will provide large-scale GWAS data in more diverse populations. For these and other studies that examine individuals of African ancestry, it is important to note that recently developed formulas for eGFR calculation that do not utilize race have become the standard in both clinical and research contexts (44).

Finally, we emphasize the need to better understand how posttranslational protein modifications impact the proteome in CKD. In one example that illustrates how posttranslational modifications can lead to divergent results, Li *et al.* (45) found that the genetic association profiles for circulating uromodulin were different depending on whether the measurement was made with aptamer-based or nucleotide-labeled antibody-based data—the identified genes suggested that aptamer measurements represented glycosylation of the protein and antibody measurements reflected abundance of the protein. In CKD research, carbamylation warrants special consideration. Carbamylation is a nonenzymatic posttranslational protein modification that results from the addition of cyanate, a dissociation product of urea nitrogen, to free amino groups (46). Carbamylated albumin levels are typically used as a proxy for overall carbamylation burden and have been found to be elevated ≥2-fold in individuals with advanced CKD relative to controls. Because this modification can change protein size, shape, charge, and function, it is a potential causal factor in CKD and its complications (47, 48). Thus, proteomic approaches that can account for this and other post translational modifications will be of significant value in CKD research.

## Conflict of interest

The authors declare no competing interests.
